# Improving user experience of SSVEP BCI through low amplitude depth and high frequency stimuli design

**DOI:** 10.1038/s41598-022-12733-0

**Published:** 2022-05-25

**Authors:** S. Ladouce, L. Darmet, J. J. Torre Tresols, S. Velut, G. Ferraro, F. Dehais

**Affiliations:** 1 Human Factors and Neuroergonomics, ISAE-SUPAERO, 31000 Toulouse, France; 2 Biomedical Engineering, Drexel University, Philadelphia, PA USA

**Keywords:** Cognitive neuroscience, Biomedical engineering

## Abstract

Steady-States Visually Evoked Potentials (SSVEP) refer to the sustained rhythmic activity observed in surface electroencephalography (EEG) in response to the presentation of repetitive visual stimuli (RVS). Due to their robustness and rapid onset, SSVEP have been widely used in Brain Computer Interfaces (BCI). However, typical SSVEP stimuli are straining to the eyes and present risks of triggering epileptic seizures. Reducing visual stimuli contrast or extending their frequency range both appear as relevant solutions to address these issues. It however remains sparsely documented how BCI performance is impacted by these features and to which extent user experience can be improved. We conducted two studies to systematically characterize the effects of frequency and amplitude depth reduction on SSVEP response. The results revealed that although high frequency stimuli improve visual comfort, their classification performance were not competitive enough to design a reliable/responsive BCI. Importantly, we found that the amplitude depth reduction of low frequency RVS is an effective solution to improve user experience while maintaining high classification performance. These findings were further validated by an online T9 SSVEP-BCI in which stimuli with 40% amplitude depth reduction achieved comparable results (>90% accuracy) to full amplitude stimuli while significantly improving user experience.

## Introduction

The Steady-States Visually Evoked Potentials (SSVEP) characterize neural responses to the presentation of periodic visual stimuli (RVS). The light information, either from Light Emitting Diodes (LED) arrays or rendered on a computer screen^1^, stimulates photoreceptor cells (cones and rods) in the retina that eventually reaches retinal ganglion cells. These cells are neurons whose activation triggers an action potentials cascade that propagates through the optic nerve to carry visual information to cortical areas. The sustained rhythmic entrainment of neuronal populations in the visual cortex to the attended RVS frequency can be recorded with surface electroencephalography (EEG). The distinctive properties of the RVS are reflected by the spectral components of the recorded EEG signal. Such effect has been widely taken advantage of to implement reactive brain computer interface (BCI). Reactive BCI are defined as the decoding of the EEG signal elicited by the presentation of a stimulus that corresponds to a specific output command. In practice, a prototypical implementation of SSVEP-based reactive BCI consists in the simultaneous presentation of several RVS varying in frequency. By focusing one’s attention to a specific stimulus, a SSVEP response is elicited and then decoded through the extraction of spatial and temporal features of the EEG signal to be fed to classification algorithms^2^. The classification of a SSVEP response triggers the associated command output (e.g., key press for a speller).

Historically, stimuli were mainly consisting in *on/off* flickering LED. Recent methods use computer screen to present flickering or pattern-reversal images (typically following a sinusoidal or square wave)^3^. The design of RVS for SSVEP-based BCI application has mainly focused on maximising the Signal-to-Noise Ratio (SNR) of the SSVEP response to achieve high classification performance. Notably, increasing amplitude depth between stimuli states ( i.e. contrast)^4^, increasing stimuli luminance^5^ or reducing user’s distance from SSVEP stimulator^6^ have proven to be successful approaches to maximise SNR. These methods are however detrimental to the user experience as they make the RVS more visually intrusive^7^. Over prolonged exposition, RVS may cause eye strain leading to visual fatigue but also reduction in task performance and headaches^3^. It should also be noted that SSVEP responses have been mainly studied within the 4 to 20Hz. This is due to a combination of hardware limitations (common monitors were limited to a 60 Hz refresh rate) and the 1/f law characterizing power distribution over the EEG spectrum. The maximal SNR for SSVEP responses has been typically observed around 15 Hz^8,9^ leading to the adoption of low frequencies stimuli for the design of SSVEP BCI. Concerns about health risks have however been raised as the presentation of high luminance RVS in the 8–20 Hz range may trigger epileptic seizures in photosensitive individuals^10^. These issues are of critical importance as they not only limit the population that can effectively use SSVEP BCI but they also imply serious health hazards for individuals with undiagnosed photosensitive epilepsy.

A first solution to address the aforementioned issues would be to use frequencies above 20 Hz in order to make RVS safer and more comfortable to the users. Previous research investigating high frequency SSVEP responses ($>20$ Hz) have mainly used LEDs displays of intense luminance. Herrmann^11^ reported SSVEP responses up to 100 Hz with a 1/f trend characterizing the magnitude of the responses^12^ and Muller et al.^13^ demonstrated the feasibility of asynchronous BCI control of a cursor using high frequency RVS (4 LEDs placed around a screen) flickering at 37 to 40 Hz. Chabuda et al.^14^ used 30 to 39 Hz RVS in a 8-class online BCI speller, reporting an average classification accuracy of 96%^15^ disclosed online classification accuracy for high frequency ranges (98.4% for 30–35 Hz, $99\%$ for 35–40 Hz and $95.2\%$ for 40–45 Hz) in a 5 class problem with LED used as SSVEP generators. However, these classification performance were only attained using long epoch length (up to 10 s) which severely limits the responsiveness of a BCI. More recent studies have applied offline analysis on 2 s epochs and Liang et al.^16^ reported 91% classification accuracy on a 40-class BCI paradigm using 30 to 36Hz RVS whereas Yue et al.^17^ reached 87% with only 1 s epoch length using 31 to 40 Hz. It is important to note that most of these studies used high luminance LED arrays placed relatively close to the participants’ eyes. This stimulation method to elicit SSVEP is arguably not suited for a daily usage, especially over prolonged use.

A second solution to improve user experience and visual comfort would be to reduce the contrast and intensity of RVS by lowering their amplitude depth. Stimulus amplitude depth refers to the contrast difference between the two antagonist states of a RVS. The mean luminance intensity is also reduced as the maximal luminance reached is lowered. In most SSVEP-based BCI implementation, the amplitude depth used is maximal. This practice is in line with findings from research on the visual system highlighting the sensitivity of primary visual cortical areas (V1) to high contrast stimuli^18^ and larger foveal magnification^19^ in response to high luminance visual information. In a recent study, Chang et al.^20^ have investigated the relevance of Amplitude Modulation (AM) for RVS to reduce eye fatigue. The AM approach consists in the modulation of the amplitude of the flickering signal by another oscillating signal of higher frequency (the carrier) over time. The authors concluded that AM, although leading to a reduction of stimuli intensity on average, was only merely perceptible to the users and did not lead to a clear improvement of visual comfort. It is important to distinguish the Amplitude Modulation approach from RVS amplitude depth reduction. Moreover, AM modulation implies an increase in the spectral complexity of the SSVEP signal, which decreases classification performance. More recently Lingelbach et al.^21^, reported SSVEP responses below an user-defined contrast perceptual threshold. The condition in which the RVS were below the perceptual threshold was rated as more visually comfortable by the participants. The RVS were not presented simultaneously but individually, thus relevance for BCI application and classification performance of stimuli below perceptual threshold remain to be assessed. In another study^22^, the authors demonstrated that a $90\%$ reduction of the maximal amplitude depth significantly improved visual comfort. The classification accuracy, although diminished in comparison to full amplitude depth RVS, was still around 80% for a 4-class problem (using a 3*s* window length). These studies sparked interest in amplitude depth reduction as a mean to improve RVS visual comfort and overall user experience during SSVEP-based BCI control.

Taken together, these findings indicate that increasing the frequency and reducing the amplitude depth of RVS are both promising approaches to make SSVEP-based BCI more comfortable and safer to use. Previous studies have however revealed that such approaches may reduce the SSVEP signal strength and therefore classification performance. Consequently, a compromise between user experience and classification performance need to be established. For this purpose we present a series of three experiments that manipulate RVS frequency and amplitude depth in a systematic manner:

- The first experiment aimed to evaluate user experience and characterize SSVEP responses elicited across 24 frequencies ranging from 8 to 60Hz (see Fig. 7);
- The second experiment aimed to investigate the effect of amplitude depth reduction (100, 50, 40, 30, 20 and 10%) on low and high frequency SSVEP responses and user experience (see Fig. 7);
- The third experiment consisted in bench-marking an online T9 BCI whose design was based on previous experiments findings. Three different RVS designs (low frequency and full amplitude vs low frequency and low amplitude depth reduction vs high frequency and subtle amplitude depth reduction).

The main ambition of the present research was to improve SSVEP-based BCI user experience. As such, subjective assessment of RVS visual comfort played a pivotal role in the decisions made regarding experimental designs across the three experiments. In addition to the measures of user experience (assessing the intrusiveness, visual comfort, and fatigue related to the presentation of RVS), SSVEP SNR and the performance of the state-of-the art classification algorithm were contrasted across the different designs.

## Results

### Experiment 1: effect of RVS frequency

This first experiment systematically compared the classification performance, subjective visual comfort and SSVEP SNR for RVS of different frequencies ranging from 8 to 60 Hz, with a step of 2 Hz.

#### User experience

**Figure 1:**
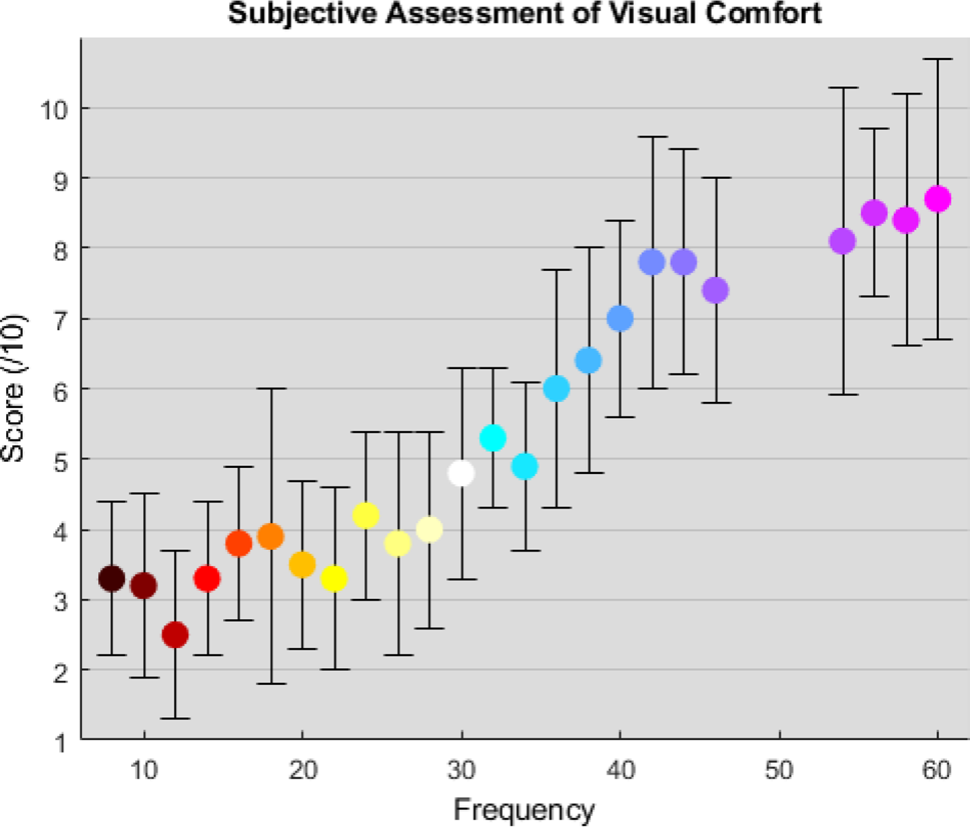
Experiment 1: relationship between RVS frequency (ranging from 8 to 60 Hz with a step increase of 2 Hz at the exclusion of 48 to 52 Hz) and the subjective visual comfort score.

The RVS frequency had a main effect on the subjective visual comfort score [F(23,264) $=$ 22.369, p < .001, $n^2$ $=$ 0.661]. There was a strong positive relationship between RVS frequency and subjective assessment of visual comfort (r(288) $=$ .781, p < .001). As illustrated in Fig. 1, a substantial difference in subjective experience was observed across the range of frequencies. The highest RVS frequency (60 Hz) are deemed much more comfortable (mean score $=$ 8.6, SD $=$ 1.9) than the lowest RVS frequency (mean score $=$ 3.3, SD $=$ 1). The subjective rating of fatigue [F(23,264) $=$ 19.035, p < .001, $n^2$ $=$ 0.624] and intrusiveness [F(23,264) $=$ 11.506, p < .001, $n^2$ $=$ 0.501] followed a similar trend with high frequency stimuli rated as less tiring and less intrusive than lower frequency RVS.

#### Critical flicker-fusion frequency

The mean flicker-fusion frequency threshold, referring to the frequency above which the RVS is not perceived as flickering, was found to be at 67.9 Hz on average (SD $=$ 2) which is in line with previous psycho-physics findings^23^. More details can be found in the Supplementary Information.

**Figure 2:**
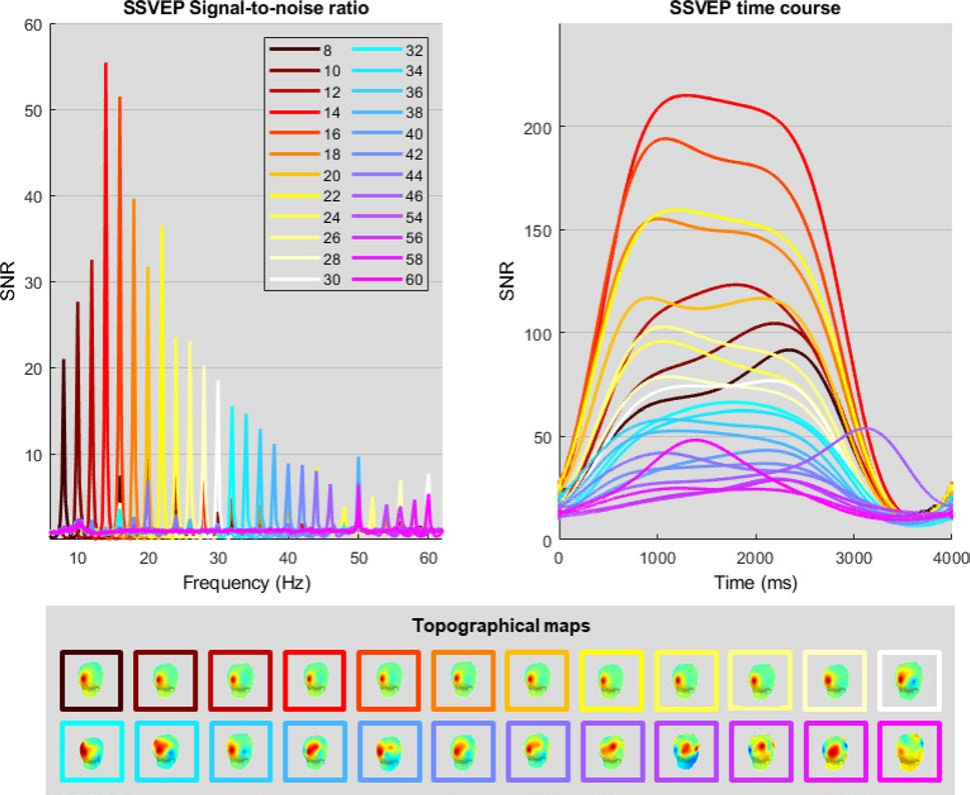
Experiment 1: rhythmic entrainment source separation (RESS)^24^ analyses of SSVEP responses to RVS ranging from 8 to 60 Hz (step increase of 2 Hz, with the exclusion of line noise neighbouring frequencies 48, 50 and 52 Hz). Top left: power spectrum of the SSVEP signal-to-noise ratio (SNR). Top right: SSVEP response over the course of the 3 s RVS presentation. Bottom: topographical distribution of SSVEP responses to RVS.

#### SSVEP signal-to-noise ratio

An ANOVA revealed a main effect of the RVS frequency on SSVEP SNR [F(23,264) $=$ 8.607, p < .001, $n^2$ $=$ 0.429]. The SNR was negatively correlated to stimulus frequency (r(288) $=$ −.692, p < .001) as can be observed in Fig. 2.

#### Classification performance

The task related component analysis (TRCA)^25^ classifier was applied to both low and high frequency data separately. Thus the classification was performed for each condition separately splitting the data into 12 classes for each condition. For the low frequencies range (from 8 to 30 Hz), we observe that the best classification performances were achieved between 10 and 15 Hz. This result is in line with previous reports from^8,9^. A steep decrease of around $20\%$ in classification accuracy was noted between the optimal frequency 14 Hz and frequencies above 20 Hz. In regards to the higher frequency range (above 30 Hz) a plateau of around $70\%$ classification accuracy was observed from 30 to 38 Hz. The breaking point was observed around 40 Hz, above which the classification performance severely declined. Additional details can be found in the confusion matrices included in the Supplementary Materials. These results are coherent with the SNR measures (see above), as SSVEP responses elicited by RVS above 40 Hz exhibited the lowest SNR values. It should be noted that this 40 Hz breaking point is far below the mean flicker fusion threshold measured at 67.9Hz. Although high frequency RVS elicited SSVEP responses, the classification performance is insufficient to ensure reliable BCI control. Furthermore, inter-subjects variability in classification accuracy increased significantly for frequencies above 36 Hz. It suggests that the highest RVS frequency that should be considered for an SSVEP-based BCI is 36 Hz.

### Experiment 2: effect of RVS amplitude depth reduction

This experiment is intended to study the impact on relative comfort and performance of reduced depth amplitude stimuli, using both high and low frequencies.

#### User experience

**Figure 3:**
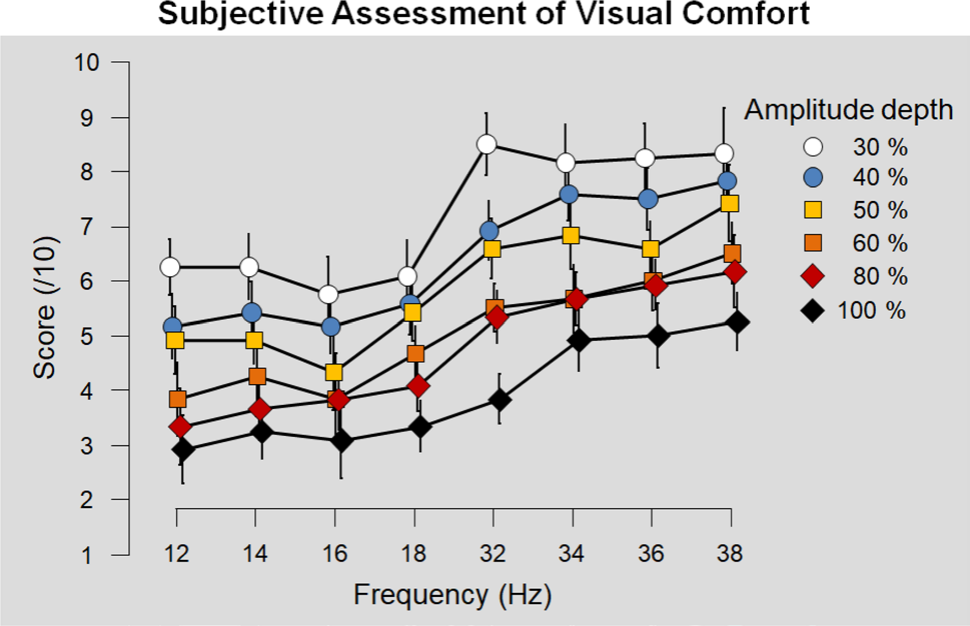
Experiment 2: subjective visual comfort score across RVS frequency ranges (low: 12, 14, 16, 18; high: 32, 34, 36, 38 Hz) for each RVS amplitude depth (30, 40, 50, 60, 80 and 100% of the maximal amplitude).

Both the RVS frequency [F(7,77) $=$ 40.88, p < .001, $n^2$ $=$ 0.339] and RVS amplitude depth [F(5,55) $=$ 196.376, p < .001, $n^2$ $=$ 0.389] had a main effect on subjective assessment of RVS visual comfort. As shown in  Fig. 3, the users rated the RVS more comfortable as their frequency increased [r(576) $=$ .573, p < .001] and their amplitude depth decreased [r(576) $=$ .602, p < .001]. The users reported that stimuli at higher frequencies induced less visual fatigue [r(576) $= -.556$, p < .001] but also that lower amplitude depth RVS reduced ocular fatigue [r(576) $= .568$, p < .001]. Regarding RVS intrusiveness, participants reported that RVS at higher frequency were less salient [r(576) $=$ 560, p < .001] and that lower amplitude RVS were also deemed as less intrusive [r(576) $=$ .528, p < .001].

#### Contrast perceptual threshold

The perception threshold of RVS following reduction of amplitude depth was assessed for each frequency individually using a staircase method (0.2 increments/decrements) for the definition of perceptual threshold. A repeated measures ANOVA with frequency (12, 14, 16, 18, 32, 34, 36, 38 Hz) as factor was performed on contrast perceptual threshold measures. The results reveal a main effect of frequency on the amplitude depth threshold at which the RVS were not perceived [F(7,77) $=$ 28,918, p < .001, $n^2$ $=$ 1]. There was a strong positive correlation between RVS frequency and the amplitude depth at which the stimuli were not perceived anymore [r(96) $= .97$, p < .001]. While lower frequencies were still perceived at around $0.8\%$ (SD $=$ 0.026) of the maximal amplitude depth, higher frequencies exhibited higher thresholds (around 3.1–4$\%$, SD $=$ 0.034). Some more details can be found in the Supplementary Information.

#### SSVEP signal-to-noise ratio

A repeated measures $6 \times 8$ ANOVA with amplitude depths (100, 80, 60, 50, 40, 30) and frequencies (12, 14, 16, 18, 32, 34, 36, 38 Hz) as factors was performed on RESS SNR measures. Both the amplitude depth [F(5,55) $=$ 3.009, p $=$ .018, $n^2$ $=$ 0.016] and RVS frequency [F(7,77) $=$ 7.883 , p < .001, $n^2$ $=$ 0.335] showed a significant effect on SSVEP SNR. There was however no significant interaction found between the two factors on SSVEP SNR [F(35,385) $=$ 0.618, p $=$ .958, $n^2$ $=$ 0.007]. A negative Pearson correlation between SSVEP SNR and RVS frequency was observed [rs(576) $= -$ .553, p < .001]. A moderate linear relationship between SSVEP SNR and RVS amplitude depth was also found [rs(576) $=$ .132, p < .01]. In brief, post-hoc comparisons revealed that lower frequencies had significantly higher SNR than high frequencies (except 32 Hz).

#### Classification performance

**Figure 4:**
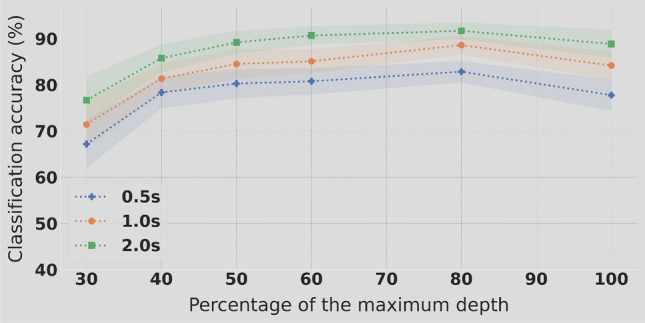
Experiment 2: classification accuracy in % using 4 classes (12, 14, 16 and 18 Hz) in function of different levels of amplitude stimulation depth and considering 3 epochs lengths.

**Figure 5:**
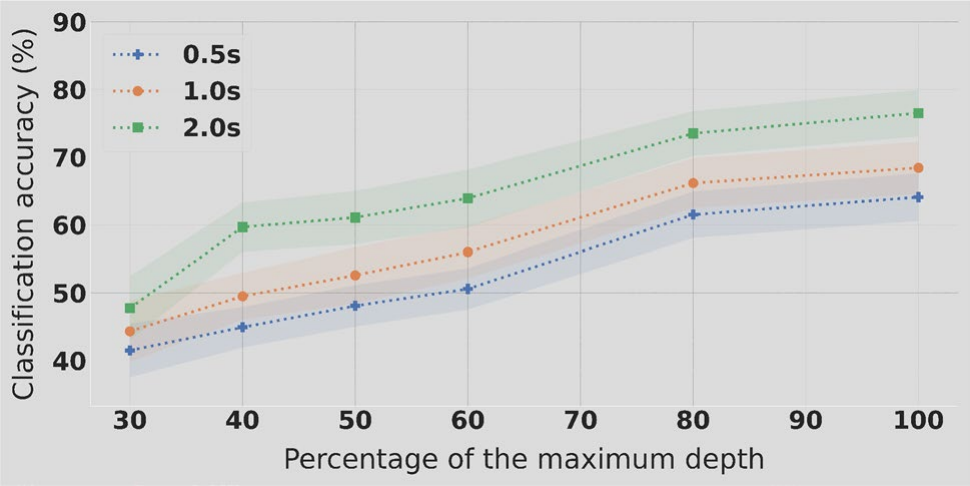
Experiment 2: classification accuracy in % using 4 classes (32, 34, 36 and 38 Hz) in function of different levels of amplitude stimulation depth and considering 3 epochs lengths.

The TRCA classifier was used to tackle a 4 classes problem corresponding to four distinct RVS frequencies. The amplitude depth reduction for the lower frequencies, 12, 14, 16 and 18 Hz, had a significant impact on classification accuracy only for amplitude depth levels below $50\%$. As can be observed on Fig. 4, above this threshold the classification accuracy estimation is higher with lower variability across individuals. Below $50\%$ of maximal amplitude depth, a significant drop in accuracy is observed. The dynamic is quite different for the higher frequencies (32, 34, 36 and 38 Hz). As depicted on Fig. 5, there is a linear trend between the increase of amplitude depth and the classification accuracy. The gain in performance is smaller between $90\%$ and $100\%$. Even though a decrease in stimuli amplitude depth is providing a better user experience, it does not seems to be operable because of the low classification performance when significant reduction (more than 30%) is considered. Only a $90\%$ reduction could be considered as the classification accuracy drop is moderated.

### Experiment 3: an online T9 experiment with improved comfort

The latter experiment involved an online setting of PIN code typing trough a T9 keyboard. Based on results of Experiment 1 and 2, we have considered a first condition using low frequencies stimuli (8–12 Hz) with $60\%$ of the maximal amplitude depth, then high frequencies (28–32 Hz) stimuli with $90\%$ of the maximal amplitude depth to finally be compared to the reference condition of low frequency (8–12 Hz) with full amplitude ($100\%$).

#### User experience

The participants were surveyed about how visually comfortable the stimuli were, how tired they felt after completing calibration plus testing phase and how frustrating the testing phase was for each condition. A main effect was found on both visual comfort [F(2,10) $=$ 53.2, p < 0.001, $n^2$ $=$ 0.914] and perceived frustration [F(2,10) $=$ 7.287, p $=$ 0.011, $n^2$ $=$ 0.593] whereas no main effect on the fatigue reported by the participants was found [F(2,10) $=$ 1.65, p $=$ 0.24, $n^2$ $=$ 0.248]. Low amplitude and high frequency stimuli were deemed as more visually comfortable than stimuli from the reference condition [reference—low amp: t(5) $=$ 10.95, p < 0.001, d $=$ 4.47, $BF_{10}$ $=$ 222.87; reference—high freq: t(5) $=$ 6.57, p $=$ 0.001, d $=$ 2.68, $BF_{10}$ $=$ 34.26]. While the user experience during the testing phase of both low frequency conditions was rated as not frustrating (Low Frequencies High Amplitude: mean $=$ 1.33, SD $=$ 1.211; Low Frequencies Low Amplitude: mean $=$ 0.833, SD $=$ 1.169), the high frequency condition induced significantly more frustration (mean $=$ 4.5, SD $=$ 3.61) [high freq—control: t(5) $=$ 2.78, p $=$ 0.04, d $=$ 1.13, $BF_{10}$ $=$ 2.5; high freq—low amp: t(5) $=$ 2.8, p $=$ 0.038, d $=$ 1.14, $BF_{10}$ $=$ 2.5]. A positive linear relationship between frustration experienced and classification performance was found for both the low amplitude [r(5) $=$ .84, p $=$.03] and high frequency [r(5) $=$ .93, p < .01] conditions. From an user experience point of view, high frequency and low amplitude stimuli were preferred by the participants as they were perceived as more visually comfortable. The experience of high frequency BCI was however deemed frustrating due to its low classification performance.

#### Classification accuracy

**Figure 6:**
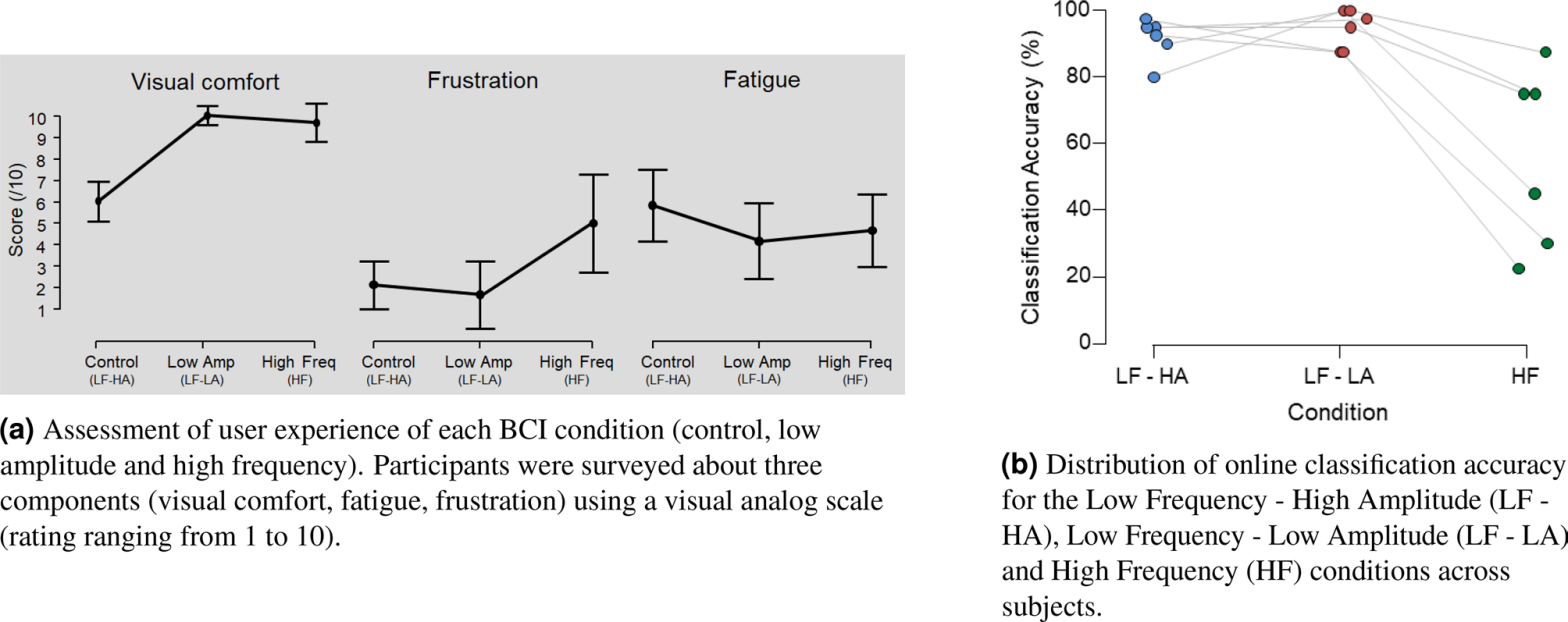
Experiment 3: subjective and classification accuracy results for the different conditions of the online T9 BCI.

The online accuracy obtained using TRCA classifier for the three conditions are summarized in Fig. 6b. The control condition (low frequencies and full amplitude) reached and accuracy of $91.7\%$ (ITR $=$ 35.9 bpm). It is to be compared with the low amplitude low frequencies condition with $94.6\%$ (ITR $=$ 38.6 bpm) and to the high frequencies conditions with mean performance of respectively with $55.8\%$ (ITR $=$ 13.0 bpm). Thus, no significant difference in terms of classification accuracy was found between the low amplitude and the control condition [t(5) $=$ .66, p $=$ .53, d $=$ .27, $BF_{10}$ $=$ .44] while performance of high frequency RVS were significantly lower. Additionally, we have observed a high variability in the inter-subjects performance for the high frequencies stimuli, as depicted by the green dots dispersion in Fig. 6b. These performances are in line with Experiment 2 findings suggesting that the use of high frequencies (>30 Hz) implies higher variability in classification performance across subjects.

## Discussion

The overarching goal of this study was to inform the design of RVS improving the user experience of SSVEP-based BCI. To this end, we firstly assessed the relevance of high frequencies (up to 60 Hz). In a second experiment we looked into the reduction of RVS amplitude depth across both low and high frequencies. Based on the results of the two previous experiments, we devised a third experiment testing different approaches for the design of comfortable stimuli with an online T9 implementation. The evaluation of an SSVEP-based BCI is usually limited to metrics such as the SNR ratio and/or classification performance. Here we propose to take an user-oriented perspective that considers visual comfort and other aspects of user experience as additional measures to evaluate the validity and usability of a reactive SSVEP-based BCI.

The first experiment disclosed that high frequencies RVS (up to 60Hz) displayed on a computer monitor could elicit measurable SSVEP responses using the RESS spatial filtering method^24^. These results are in line with findings from previous studies^11,26^. These previous studies used LEDs for the stimulation presentation which typically produce more luminosity compared to our computer screen. We used a computer screen as it offers higher flexibility for the design of reactive BCI. Solely in regards of the user-focused approach, high frequencies SSVEP appeared to be the most comfortable stimuli. Moreover, they are more likely to reduce the risks of epileptic seizure and therefore are theoretically a good idea. However, in practice the classification performances (only $54\%$ on average for the online T9) are not substantial enough. This downgrading of performance can be seen as consequence of the 1/f trend for the magnitude of brain responses demonstrated in^11^. It should also be noted that the inter-subjects variability is large. Three subjects out of six have achieved satisfactory results ($> 78\%$) and the three others are below $50\%$. Another limitation of high frequencies stimuli is that they are less perceptible and called for more focus and concentration. In brief, we have been able to decode elicited brain responses from high frequencies (>30 Hz), as previous studies, but this was not sufficient for a reliable control of the online BCI. In^27^, the authors have designed a neural network to decode the pattern of visual stimulation. This decoding is used in a second step to control a BCI. They have reported high variability between subjects in decoding performance for the first step, while the inter-individual differences for the control of the BCI were way lower. Thus, the capacity of decoding brain signal does not automatically and directly translate into powerful BCI control availability. On another note, high frequency SSVEP could be relevant to improve the visual comfort for basic SSVEP-based psycho-physics experiments, where usually multiple trials are averaged for post-hoc analysis and do not require single trial classification.

Our second and third experiment disclosed that the reduction of amplitude depth offers the best compromise in terms of classification performance and user experience (see Fig. 6a), as long as low frequency are considered. Importantly we report that a substantial reduction of amplitude depth, up to $50\%$, did not lead to a significant decrease of classification performance while improving the user comfort. Therefore it should become a standard and always consider. It should be noted that the use of high frequencies stimuli require the use of specific hardware, screens with high refresh rate while the amplitude depth reduction does not imply a more complex implementation or changes in the hardware. A larger reduction of stimulation amplitude depth could also be considered when visual comfort and fatigue become crucial, for example when the operating time is long. However, it would be at the expense of a small decrease in classification performance.

On another note, the classification speed, trough Information Transfer Rate (ITR) classically mainly, is emphasized metrics to evaluate BCIs performance. The computation of the ITR takes into account the number of targets *N*, the classification accuracy *P*, the total number of trials *S* and *T* the length of the experience. In our case, the BCI provides an ITR of 38.6bpm on average for the control condition which can be low in comparison to recent studies^25^. One reason, is that our design includes an inter-trial of 2.5 s to allow the subject to check the output of last step and acknowledge the next figure to type. We could have asked our participant to memorize the four digits to type or have them cued and removed the classification feedback to have a reduced inter-trial of 0.2 s. It would not have impacted the classification accuracy and provided an increased ITR of 76.7 bpm which compares favorably to a benchmark study^28^. However, the user experience would have been drastically different with less sense of control. This shows a clear limit of the metric. As discussed in^27^, a trial duration (stimuli plus pause between trials) below 1 s makes the system too fast to be used by an untrained user. Beyond a certain performance threshold, improving the ITR would be usually at the expense of the user experience: trials too short for good control, increased number of errors, absence or limited feedback, etc.. These considerations about the limitations of BCI performance measures^29^, especially ITR, advocate in favor of considering a trade-off between classification performance and user experience to evaluate the general performance of a BCI.

In the presented experiments, visual comfort, fatigue and frustration was assessed through self-reported questionnaires. This subjective approach allows for a direct quantification of user experience with minimal cost time and effort wise. While this approach is practical for the systematic evaluation of user experience over a wide range of stimuli, as reported here, self-report questionnaires are however subject to biases. It can therefore be argued that the acquisition of objective measures would be desirable to further validate and strengthen the present findings. As such, eye-tracking and pupillometry metrics offer valuable insights on the visual comfort and fatigue experienced by the user. Future works focusing on human factors and user experience of VEP-based BCI would benefit from including such objective measures.

To conclude, our study disclosed that the reduction of amplitude depth presented the best compromise in terms of user comfort, frustration and classification score compared to using high frequency SSVEP. We hope that this work will foster research efforts to study the effect of other stimuli features. Checkerboards have been considered^30,31^ to increase the elicited brain response^32^. But in the perspective of an SSVEP-BCI, they have never been studied systematically along other textures. Instead of flickering, spatial oscillations of the flickers could also be evaluated. Beside that and still to provide better ergonomic for SSVEP-BCI system, it could be beneficial to have a gating process, such using an eye-tracker or a physical button, to make the stimuli flicker only when the user intend to interact with it. In a realistic use case, a continuous flickering would be tiring and even distracting when operating other tasks. Eventually, another bottleneck of the large use of SSVEP-BCI outside our laboratories is the hardware. In this study, we have relied on a classical wet-EEG setup. It remains tedious to equip the subject and subject should wash his air after the use. Existing hardware of dry-EEG still induce drop of accuracy compared to wet-EEG^33^ and the comfort is not optimal^34^. Nevertheless, it is an important and promising approach to improve the user experience of SSVEP BCI.

## Methods

### Participants

A total of thirty subjects took part in the study. For the first experiment focusing on the exploration of RVS frequency, 12 participants were recruited (mean age = 26, SD = 6, 8 male and 4 female). The second experiment investigating reduction of amplitude depth was performed by 12 participants (mean age = 27, SD = 5, 8 male and 4 female). The online T9 digicode BCI was tested on 6 individuals (mean age = 2, 8 SD = 4, 5 male and 1 female). The participants did not report any of the exclusion criteria (neurological antecedents, usage of psychoactive medication). The study was approved by the ethics committee of the University of Toulouse (CER approval number 2020-334) and was carried in accordance with the declaration of Helsinki. Participants gave informed written consent prior to the experiments.

### Stimuli and procedure

One of the main challenge of SSVEP-based BCI paradigms relates to achieving precise and reliable presentation of RVS. This is especially true for the elicitation of high frequency SSVEP responses. Due to the Nyquist-Shannon sampling theorem, the highest frequency that can be presented is equal to half of the monitor maximal refresh rate. For this reason, most studies that have reported SSVEP responses above 20 Hz have used LEDs as SSVEP generator method. LED arrays as a SSVEP generator offer less flexibility for the design of comfortable RVS. Indeed, any modification of features (e.g., position, colour, size) of the LED array implies hardware replacement to be performed manually. The emergence of high frequency monitors (144 and 240 Hz) contributed to drastically extend the range of frequency that can be reliably displayed on computer monitors. Capitalizing on this technical advance, we were able to readily manipulate features of visual stimuli presented at high frequency on a computer monitor.

The stimulus presentation program was written in *C* for the two first experiments and in Python using Psychopy2^35^ for the online T9 experiment. A lower-level programming language, *C* was preferred for the first experiment as it allowed a faster computation for the presentation of stimuli at high frequencies (above 50 Hz). This was not required for the online experiment as the frequencies selected only went up to 35 Hz. The code used for the online classification is written in Python. The Python code of the T9 graphical interface and the classification is available online (https://github.com/ludovicdmt/t9_ssvep). Classification was performed on EEG data and marker streams received trough LSL^36^ from the stimulus presentation program. The stimuli were presented using a sampled sinusoidal on a 27-inch LCD monitor (1*ms* IPS display, *NVIDIA G-sync* compatible), a resolution of $1920\times 1080$ pixel (width $\times$ height) and a luminance of 400 cd $m^{-2}$. The refresh rate was set to 240 Hz to present high frequencies with high precision. For the two first experiments stimuli were rendered within a $213\times 213$ pixels rectangle centered on the screen. For the T9 experiment, each square was of size $150 \times 150$, with a margin of 250 pixels between each square. For the first two experiments, RVS stimuli were presented on top of a grey background whose luminance was of 124 lux (measured using a digital light meter from Extech Instruments). For the online BCI implementation, the dark background used had a luminance of 60 lux.

**Figure 7:**
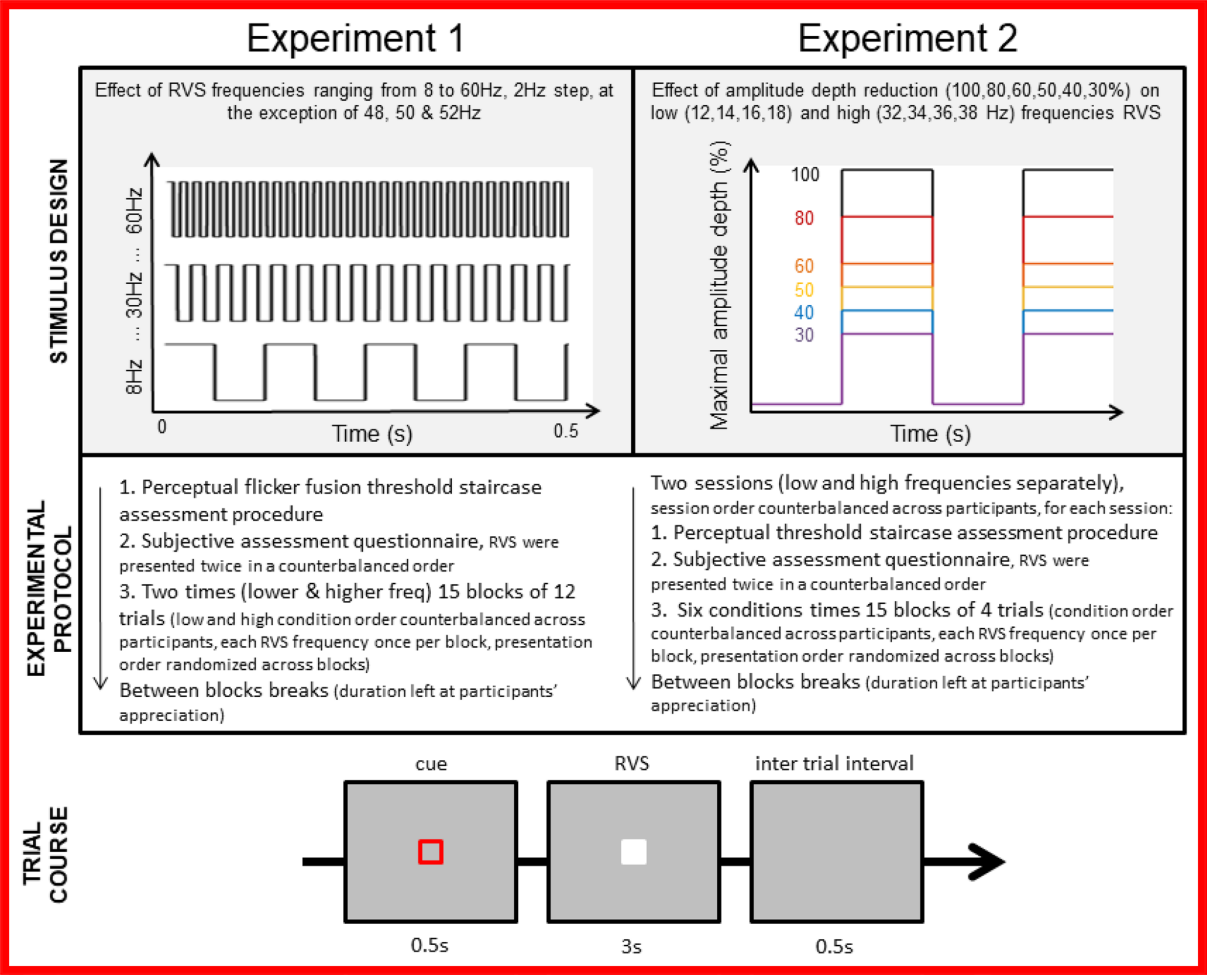
Experiments 1 and 2 : illustration of the repeated visual stimuli (RVS) design for each experiment (manipulating stimuli frequency and amplitude depth), experimental protocols descriptions and a diagram presenting trial course parameters used across both experiments.

#### Experiment 1: high against low frequencies with full amplitude of simulation

In this first experiment we investigated the subjective experience and SSVEP in response to stimuli of a wide range of frequencies. The subjects underwent a single session that was cut in two separated parts. Firstly, we have presented sequentially and individually stimuli with twelve, low, frequencies from 8 to 30 Hz with a range of 2 Hz and then another 12, high, frequencies from 32 to 60 with a 2 Hz range (48, 50, 52 Hz were excluded because of power line noise). Half of the subjects started the session with low frequencies and the other half with high ones. Each session started with the presentation of individual flickers, low and high frequencies, alone and with a pseudo-random order, so as to fill the subjective assessment questionnaire of visual comfort and the fusion flicker frequency. Participants were asked to grade each stimulation with a mark from 1 (uncomfortable) to 10 (comfortable). We also have presented flickers above 60Hz and asked the volunteers to acknowledge when they do not perceive the flickering anymore (fusion flicker effect). After this qualitative assessment phase, for both high and low frequencies, there were 15 blocks with one trial per class to quantitatively study the flickers. The presentation order of the frequencies inside each block was pseudo-random. Each stimulation was preceded by a red circling, visual cue, for 0.5 s, it then lasted for 3 s, followed by an inter-trial of 0.5 s. The 3 s epochs were then cut offline to accommodate for different trials lengths during analysis. Between each block their was a pause screen and subject had to press space bar when he was ready for the next block. A graphical summary of the experimental protocol and trial course can be found in Fig. 7. The total length for one session, including the setup of the wet-EEG and the questionnaire, was less than one hour. As in this currently the state of the art classifier for synchronous SSVEP, we have used Task Related Component Analysis (TRCA)^25^ as classifier in the offline analysis. Further details on the classifier are provided in the following methods sections (page 11) and in the Supplementary Information. For both high and low frequencies condition, the background was set to gray and flickers oscillated between gray and white. Twelve healthy individuals (4 female, mean age = 26, SD = 6 SD) took part in the single session of this experiment.

#### Experiment 2: reducing amplitude depth of simulation

In the second experiment, our aim was to explore a reduction in the amplitude depth of stimulation for both high and low frequencies stimuli. We have quantitatively and qualitatively studied the trade-off between accuracy and user comfort for reduced amplitude stimuli. To the best of authors knowledge, while the use of a broader range of frequencies was studied, mostly with LED, a reduction of stimulation amplitude to control a BCI was only consider in a preliminary quantitative study^22^. Consistent with the conclusions of the Experiment 1, we have selected 12, 14, 16 and 18 Hz as for the low frequencies condition and 32, 34, 36 and 38 Hz for the high frequencies. The number of trials and stimulus presentation duration was kept consistent with Experiment 1: 15 trials, 0.5 s of cue, 3 s stimulation and inter trial of 0.5 s with a self-paced break (space bar press to continue) between blocks. Prior to the experiment, the perceptual threshold for the minimal amplitude depth was assessed through a staircase procedure (starting from 5% of the maximal amplitude depth with decrements of 0.1%). Then the session started with the subjective assessment of each flicker with the different amplitude depths, before switching to the blocks presentation and EEG recording. The amplitude depth was manipulated across six levels ($100\%$, $80\%$, $60\%$, $50\%$, $40\%$, $30\%$). The definition of the range was so to achieve good sensitivity from 30 up to $100\%$, with lowest amplitude depths being doubled (30, 40 and 50 with the corresponding 60, 80 and $100\%$). The total duration of Experiment 2 was twice as long as Experiment 1. It was therefore split in two sessions to avoid fatigue effects. The order of conditions was also counterbalanced across participants. The two sessions took place on different days and were separated by a maximum of 1 week. The experimental protocol and trial course are depicted in Fig. 7.

In practice the implementation of the amplitude depth reduction used a sinusoidal sampled to the refresh rate of the screen. The background was set to gray as in Experiment 1. The RVS were oscillating from gray (value of 130 on the gray scale) to white (value of 255 on the gray scale) for full amplitude and from gray to lighter shades of gray for the amplitude depth reduction RVS. For instance, a RVS with reduced amplitude of $40\%$ would oscillate between 130 and $130 + (255-130) \times 0.4 = 180$ on the gray scale. The maximum depth reduction that allows perception of RVS contrast change was also an open question which was investigated in Experiment 2. The rationale for and the definition of this perceptual threshold is comparable to the fusion-flicker threshold procedure described in Experiment 1. We used TRCA as in Experiment 1 for the offline classification analysis. Twelve healthy individuals (4 female, mean age $=$ 27, SD $=$ 5.25) took part in both sessions of this experiment. Four participants had already taken part to the first experiment.

#### Experiment 3: an online T9 experiment with improved visual comfort

This third experiment was designed to test the validity of high frequency and low amplitude stimuli in the context of a realistic SSVEP-based BCI. Based on the previous findings from experiments 1 and 2, we formulated the hypothesis that high frequency and low amplitude RVS can improve user experience while maintaining high classification performances for BCI control. This study also aimed to investigate the limitations of high frequency stimuli in an online BCI context. The classification was therefore performed online and direct feedback was provided to the user.

The BCI consists of a T9 keyboard, smartphone keyboard, with numbers from 0 to 9, and *Back* (11 classes problem). We have asked the participants to type 10 codes of 4 numbers, PIN codes. The specified PIN code was displayed on the left of the T9 and the output of the BCI on the left. You can find a screen-shot of the interface in the Supplementary Information. In addition to the visual feedback on the right of the screen, an auditory feedback was provided with a low-pitch tone for an error of classification and a high-pitch tone for a correct classification. Background color was set to black to further improve contrast with the RVS.

The following three conditions were tested. High frequencies (30–34.6 Hz) and slightly reduced amplitude ($90\%$) stimuli [HF]. Low frequencies (10–14.6 Hz) and largely reduced amplitude depth ($50\%$) [LF-LA]. And Low frequencies (10–14.6 Hz) with full amplitude depth [LF-HA] as a control condition reflecting the classical RVS design used in previous SSVEP-based BCI.

To display the 11 classes within a reduced frequency range of 4.6 Hz, we took advantage of the Joint Frequency and Phase Modulation (JFPM)^37^, as used in the original paper of the state of the art method TRCA^25^. The RVS frequency were vertically spaced of 0.2 Hz with phases differences of $+0.35\pi$ and horizontally spaced with 2 Hz with phases differences of $-0.5\pi$, using sine waves. This implementation is schematized in the Supplementary Information. The RVS were oscillating between black and white. Similarly to previous experiments, we have used the TRCA classifier^25^. Trial length was set to 2.0*s* to maximize accuracy over ITR, as discussed on 7. In order to ensure a reliable classification accuracy, 12 calibration samples were collected for each class. This approach is comparable to the original TRCA study^25^ that used 10 calibration sample per class. The calibration was divided in 12 blocks, within which each class was presented as a target once. The presentation order was pseudo-randomized and different for each block. One of the stimulus was cued for 0.5 s with a red circling. Then, the stimulation lasted for 2.13 s and only the last 2.0 s were kept. The first 0.13 s of the stimulation period were discarded to account for a latency delay of information going through the visual pathway^38^. Finally, a pause of 0.7 s as inter-trial was completed. During the online test phase, the inter-trial was set to 2.5 s to allow the user to check between each trial the specified PIN code on the left and output from previous commands on the right. We could have reduced the inter-trial time to increase the ITR but our aim was to have a user-friendly interface that allows realistic interactions with the BCI. Stimulation was also set to 2*s* during the test phase. The pause duration between calibration blocks and the input of 4 consecutive digits PIN code was left to participants’ appreciation (space bar had to be pressed to continue the experiment). Six healthy individuals (1 female, 7 males, mean age = 28, SD = 4) took part in the single session of this experiment.

### EEG data acquisition

EEG data was recorded from 32 electrodes fitted in an elastic cap according to the 10–20 international system and connected to a LiveAmp amplifier (Brain Products, Munich, Germany). The ground electrode was placed at the Fpz electrode location and all electrodes were referenced to FCz electrode. The electrode impedance were brought below $20k\Omega$ prior recording. The signal was acquired at a rate of 500 Hz with a digital band-pass filter ranging from 0.1 to 250 Hz. At the onset of every stimulus presentation, an event trigger was generated by the stimulus presentation program and synchronized to the EEG data stream via Lab Streaming Layer (LSL)^36^. Eventually, only height electrodes (O1, O2, Oz, P3, P4, Pz, P7, P8) are manually selected to perform classification while all electrodes are used for Fourier frequency analyses. The manual selection of electrodes was centered around the occipital region. The raw data from the three experiments, with all electrodes included, are available online https://zenodo.org/record/5907009.

### Subjective tests

In each experiment, questionnaires assessing participants’ subjective experience of RVS were administered to gather information about how visually comfortable, tiring and intrusive the stimuli were deemed. For this, RVS were presented for three seconds to the participants. Then the participants rated the RVS in terms of the three aforementioned dimensions (visual comfort, fatigue, intrusiveness) by assessing them on analog scales ranging from 1 to 10. This procedure was repeated for each condition of the corresponding experimental design. The presentation order of the RVS within these subjective assessment trials was randomized across participants.

### SSVEP analysis

Rhythmic Entrainment Source Separation (RESS) algorithm^24^ is a supervised source separation technique. It was use to asses in an offline manner the signal quality and characteristics from the Experiment 1 and 2. It computes a spatial filter *w* that is intend to extract the signal of interest elicited by the SSVEP. The filter *w* maximizes the covariance matrix of data filtered, narrowly, around the SSVEP frequency of interest while minimizing covariance matrix out of the narrow band. The SSVEP activity is supposed spatially stationary over time and thus *w* is computed only one time for all trials.

### Classification algorithm

We have selected Task-Related Component Analysis (TRCA)^25^ algorithm that is state of the art for SSVEP to assess classification performance with regards to the different stimuli designs. TRCA model achieved more than $90\%$ accuracy and Information Transfer Rate (ITR) of more than 300 bits/min on a 40 classes Brain Speller, using 0.5s epochs. It has been since largely endorsed by the community, even using dry electrodes^33^. The others classical classification algorithms for SSVEP are based on Canonical Correlation Analysis (CCA)^39,40^, with multiple nuance to define EEG template to compare with. In^25^, these CCA-based methods provided significantly lower results compared to TRCA. Therefore, we chose to compute results only with the TRCA method. It would represent classical, generic and state of the art framework for SSVEP classification. Beside that, some incremental gains of performance could be provided by more calibration data but it would not have been in favor of a better user experience. More details on the TRCA algorithm can be found in the Supplementary Information. In our study, we observed better results when applying a downsampling of factor 2 to have a sampling rate of 250Hz, as is the original paper of TRCA. We used a Python implementation by our team (https://github.com/nbara/python-meegkit/blob/master/meegkit/trca.py), based on the the original Matlab code shared by the authors, that can be found here (https://github.com/mnakanishi/TRCA-SSVEP).

## Supplementary Information

Supplementary Information.

## Data Availability

The datasets generated and analysed during the current study are available in the Zenodo repository, https://zenodo.org/record/5907009.
