# Supplementary material for: Improving user experience of SSVEP BCI through low amplitude depth and high frequency stimuli design
Source: Sci Rep. 2022 May 25;12:8865. doi: 10.1038/s41598-022-12733-0 (PMC9132909; doi:10.1038/s41598-022-12733-0)
Supplement: Supplementary file 1 — Supplementary Information. [file 41598_2022_12733_MOESM1_ESM.pdf]

Supplementary Information for

Improving user experience of SSVEP BCI through low amplitude depth and high frequency stimuli design

S. Ladouce, L. Darmet, J.J.T. Tresols, S. Velut, G. Ferraro and F.Dehais

Corresponding Author : simon.ladouceisae-supaero.fr

This PDF file includes:

Supplementary Method
Supplementary Figures 1 to 5

Supporting Information Method

Task-Related Component Analysis (TRCA). TRCA is a template-matching based method. It has been originally developed for functional near-infrared spectroscopy (fNIRS) data and applied to SSVEP-BCI and EEG by Nakanishi *et al.*. TRCA uses calibration data to compute an individual template and a spatial filters that are subject-dependant. The spatial filters maximise the inter-trial correlation within each class, while keeping a bounded variance:

$$\widehat{w_f} = \arg\max_{w} \frac{w^T \mathbf{S_f} w}{w^T \mathbf{Q_f} w}$$

with $\widehat{w}$ the obtained spatial filter for frequency f, Q the covariance of the concatenation of all trials of frequency f and $\mathbf{S}$ is the sum of all covariances between separate trials for that frequency.
Then spatially filtered data are matched with individual template defined during calibration to estimate the SSVEP frequency of a test epoch. In addition to these steps a filter-bank approach is conducted. Thus the following steps are repeated across m bands obtained with the filter bank. The upper bounds of the filters in the filterbank are of 90Hz, as in (**?**). In practice we have noticed that there is little EEG brain signal above 90Hz, it is mostly noise. The lower bounds are so that each band contains one harmonic of the SSVEP stimuli less than the previous band. For instance with a 4 classes problem with stimuli of 12, 14, 16 and 18Hz, lower bound for the first band is 12, then 24Hz, then 36Hz, 48Hz and so. The maximal number of band used is $m = 5$ as in original work of Nakanishi *et al.*. It is only $m = 2$ for higher frequencies such as 30Hz (*i.e* 30 and 60Hz).

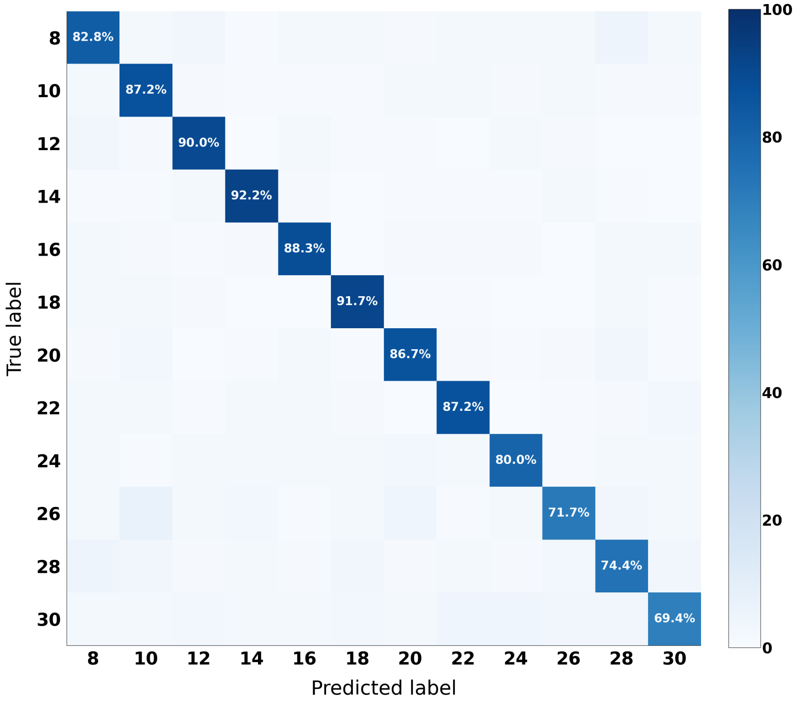

Supplementary Figure 1. Experiment 1: Normalized confusion matrix averaged over all subjects for low frequencies (8-30Hz) using TRCA classifier and 1.0s epoch. It is a 12 classes problem. Values outside of the diagonal are all $< 10\%$

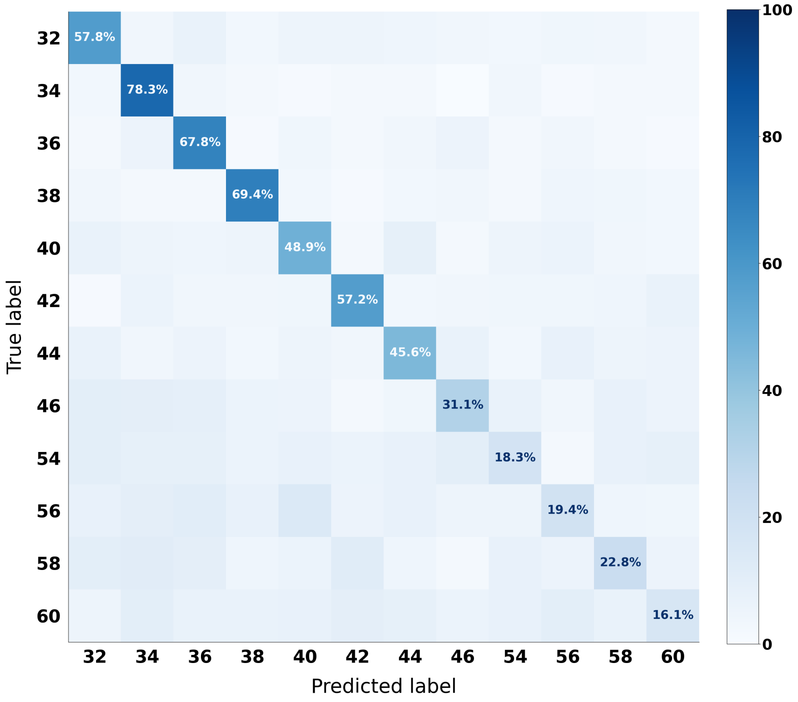

Supplementary Figure 2. Experiment 1: Normalized confusion matrix averaged over all subjects for high frequencies (32-60Hz) using TRCA classifier and 1.0s epoch. It is a 12 classes problem. Values outside of the diagonal are all $< 10\%$.

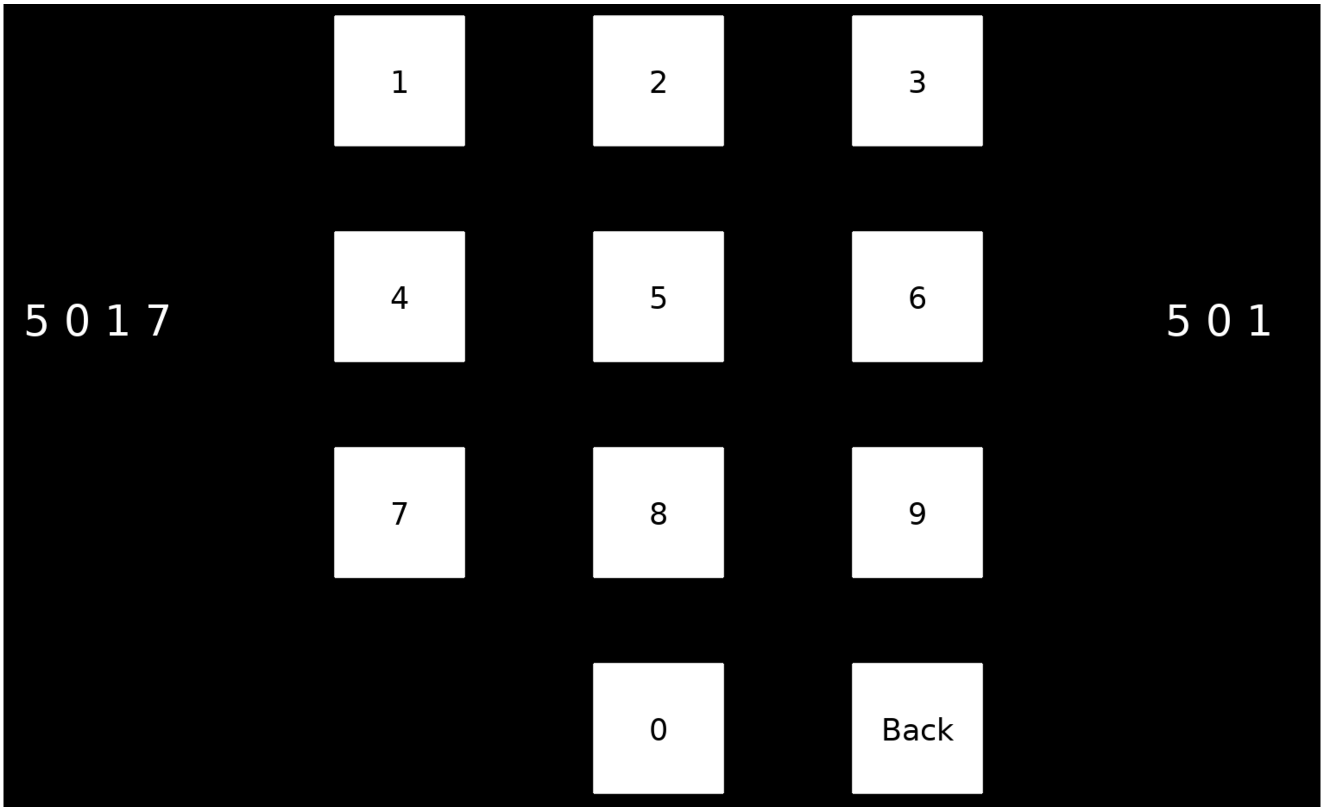

Supplementary Figure 3. Experiment 3: Graphical interface of the online T9 SSVEP BCI. Specified code is on the left and the output of the classifier on the right (here after three correct predictions)

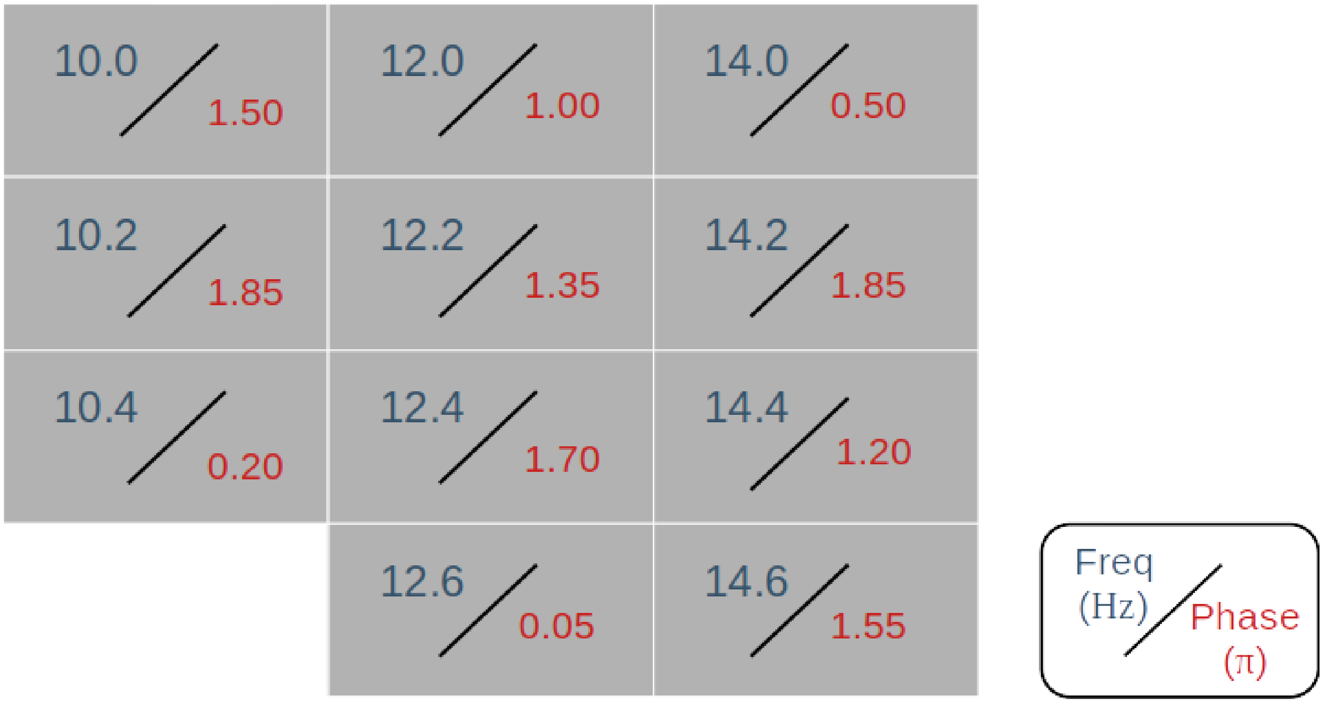

Supplementary Figure 4. Experiment 3: Frequencies and phases for the 11 classes of the T9. Phases are times π, for instance 1.50 means 1.5π. For the high frequencies, a +20Hz is added to the frequencies, without any change to the phases.

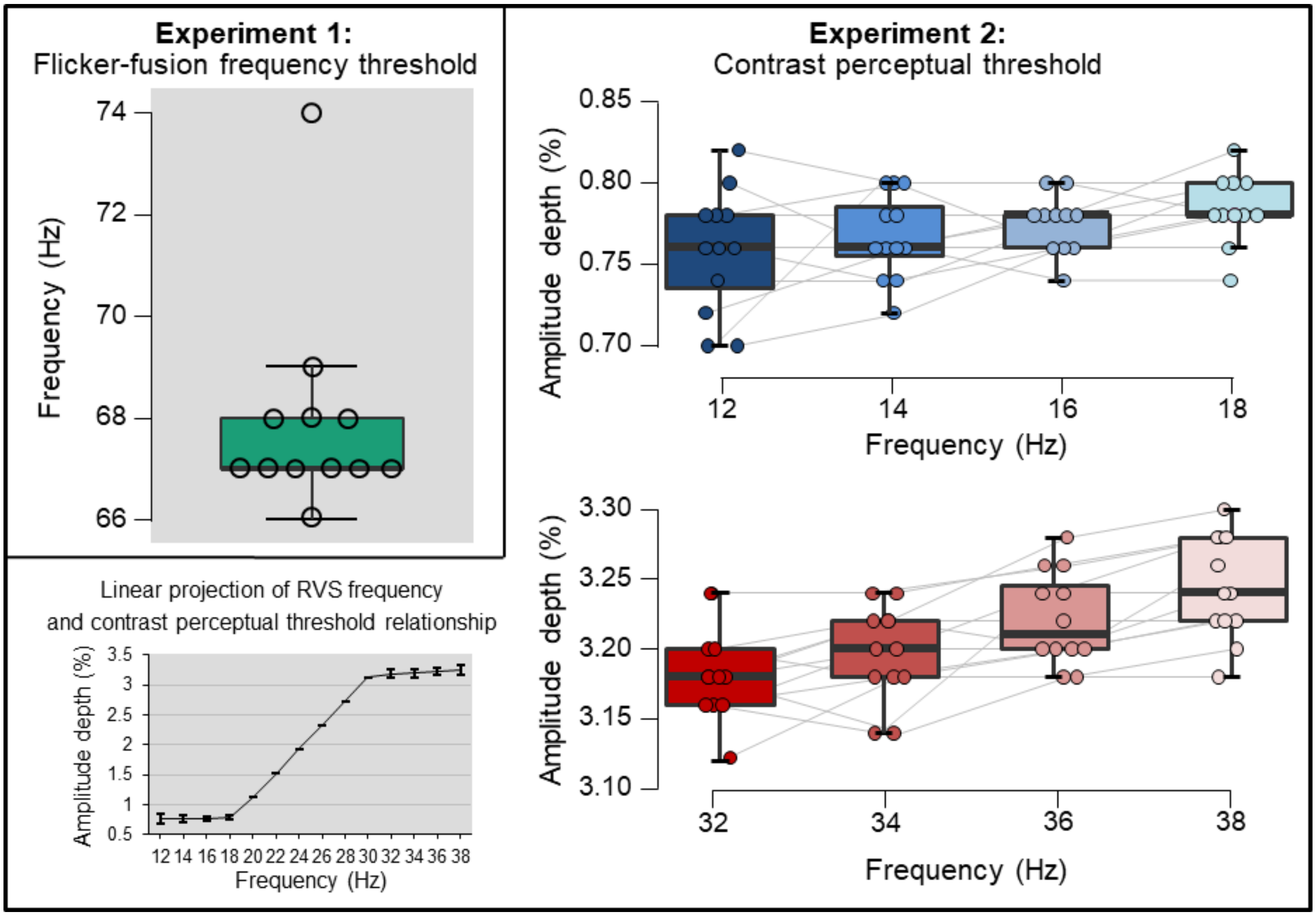

Supplementary Figure 5. Measures of critical flicker fusion frequency (Experiment 1) and contrast perceptual threshold (Experiment 2) using a psychophysics staircase protocol. Top left: Distribution of the maximal frequency at which the RVS was perceived as flickering across participants. Right side: Distribution of the minimal amplitude depth required for the perception of contrast change across frequencies (lower frequency range on top and higher frequency range at the bottom). Left bottom: Estimation of the relationship between RVS frequency and contrast perceptual threshold using linear interpolation.
